# Thermal Degradation of Polystyrene (PS) Nanocomposites Loaded with Sol Gel-Synthesized ZnO Nanorods

**DOI:** 10.3390/polym12091935

**Published:** 2020-08-27

**Authors:** Ashraf H. Farha, Abdullah F. Al Naim, Shehab A. Mansour

**Affiliations:** 1 Department of Physics, College of Science, King Faisal University, P.O. Box 400, Al-Ahsa 31982, Saudi Arabia; anaim2@kfu.edu.sa; 2 Semiconductors Technology Lab, Physics Department, Faculty of Science, Ain Shams University, Cairo 11566, Egypt; 3 Advanced Materials/Solar Energy and Environmental Sustainability (AMSEES) Laboratory, Faculty of Engineering, Menoufia University, Shebin El-Kom 32511, Egypt; shehab_mansour@yahoo.com

**Keywords:** ZnO nanoparticles, polystyrene PS, chemical synthesis, polymer nanocomposite, sol-gel, DSC, thermal properties

## Abstract

Thermal degradation of polystyrene/ZnO (PS/ZnO) nanocomposites was investigated in this study. PS/ZnO polymer nanocomposites were prepared by using ZnO nanorods as nanofillers that were prepared via the sol-gel route. The as-prepared ZnO nanoparticles showed nanocrystallites in rod-like shapes with a non-uniform hexagonal cross-section and diameter varying from 40 to 75 nm. PS/ZnO nanocomposites with ZnO nanoparticles content ranging from 0–3 wt% are prepared via the common casting method. Even dispersion for ZnO nanoparticles within as-prepared PS/ZnO nanocomposites was verified through SEM/EDX measurements. Thermal degradation of the samples was checked by using the thermogravimetric (TG) analysis and differential scanning calorimetry (DSC) under non-isothermal conditions and a constant heating rate of 10 °C min. The thermal stability of the nanocomposite is elevated compared to that of pristine PS due to the addition of the ZnO nanoparticles. The homogeneity of the PS/ZnO nanocomposites is verified by systematic increases in thermal degradation with increasing ZnO content. The characterization degradation temperatures at different weight loss percentages of ZnO nanoparticles increase at high ZnO wt%. Static activation energy of decomposing is based on TGA data. Activation energies showed some enhancement after the addition of ZnO nanorods into the PS matrix. Enhancing the thermal stability of PS with ZnO addition within the investigated ZnO concentration range is verified by TG, DSC results.

## 1. Introduction

Functional materials with improved electrical, mechanical, and thermal properties have received a lot of attention recently [1,2]. Polymer nanocomposites, which result from combining both organic and inorganic materials, are one of the important groups of functional materials. The introduction of nanoparticles with specific amounts within the polymer matrix results in improvements in many of the conventional physical properties, as well as showing synergistic effects on the properties of host polymers [3,4,5]. Such types of organic and inorganic materials combinations are resulting in new material(s) with high potential usage in broad types of applications [1,2,3,4,5,6,7]. The resulting improvements in the properties of the polymers include excellent mechanical durability, thermal stability, ease of processing, molding, and chemical resistance to reagents [5,7].

The aims of improving the properties of both components of the new combinations are because of their demand in many technological applications. Several properties of the polymers have been tuned by proper type and/or amount of nanoparticles that were used as polymers fillers. The incorporation of the inorganic nanomaterials such as ZnO nanoparticles into the organic polymer matrix resulted in polymer/ZnO nanocomposites that had excellent magnetic, mechanical and high thermal stability properties [5,8,9,10]. Enhancing the thermal stability of the nanocomposites is a crucial aspect for many of their industrial applications [7,11,12,13].

In general, the great attention that has been paid to the polymers in recent years was because of their important applications in different fields. Polymers are used as sensors, light-emitting diodes, and various solar applications [14,15]. One of the interesting polymers that received a lot of this attention is polystyrene (PS). PS attracted attention due to its interesting optical and superior thermal and chemical stability features [15]. The good properties of PS included high optical transparency, very good electrical insulation, high thermal resistance, low density, excellent mechanical durability, and convenience of processing and molding [5,10,14,15,16]. All these advantages are making it an essential element in lots of market applications such as product packaging, extruded sheets and electronics [17,18]. Although it has all of these previous advantages, still there are some shortcomings, such as its high flammability and severe dripping during combustion [9,19]. Some means were proposed for overcoming such shortcomings. One way is blending PS with one or two or more polymers with different physical properties for enhancing the properties of resulting polymer materials [6,20]. Another way to overcome these shortcomings of PS is by attaining PS-new materials with enhanced properties through the nanocomposites approach. Most of the research studies that have been done to improve the thermal stability of PS polymer were done by adding different types of inorganic nanoparticles as fillers [21]. PS nanocomposites with carbon nanoparticles fillers were reported [5,10,15,22]. Among the inorganic fillers that have been added into the PS matrix are ZnO nanoparticles. Wacharawichanant et al. [6] reported no effect of the addition of ZnO of glass transition temperature T_g_ of PS/ZnO blends with other polymers and they also reported improvements in the thermal degradation of the blends as ZnO content increased. Other work on Ps/ZnO nanocomposites, for only one composition at ZnO of 5 wt%, reported an increase of T_g_ as ZnO content increased in the sample [23]. Other work showed that the introduction of commercial ZnO nanoparticles into the PS matrix causes a very slightly increase in T_g_ [10]. Most of these previous works were done at specific concentration of ZnO or in a smaller range. No such work was reported on ZnO nanoparticles with rod-like shapes as nanofillers. The aim of this study is to check the effect of the addition of ZnO nanoparticles with rod-like shapes of concentrations (0, 0.5, 0.7, 1.0, and 3.0 ZnO wt%) on the thermal stabilization of the PS matrix. The thermal properties and thermal stability of the under-investigation samples were checked and reported. Thermal degradation mechanisms of PS/ZnO nanocomposites were also studied here.

## 2. Experimental

### 2.1. Materials

Polystyrene (PS) pellets provided by (Sigma-Aldrich, St. Louis, MO, USA) were used. N,Ndimethylformamide (DMF, (Alfa Aesar, Haverhill, MA, USA)) was used for the preparation of PS/ZnO nanocomposites. ZnO nanocrystals in rod-like shapes that were synthesized using sol-gel route as reported elsewhere [24,25] were used as nanofillers. In such a typical route, zinc acetate dehydrate (Zn(CH_3_COO)_2_.H2O), isopropanol and diethanolamine (HN(CH_2_CH_2_OH)_2_, DEA) (Sigma-Aldrich, St. Louis, MO, USA) were used as salt precursor, solvent and chelating agent, respectively.

### 2.2. Preparation of PS/ZnO Nanocomposites

PS/ZnO nanocomposites were produced by a simple solution casting method. The desired amount of PS was added to 20 mL of DMF and stirring until completely dissolved. Then the proper amount of ZnO nanocrystals powder was added to the solution with stirring and kept for 2 h at room temperature (RT). The obtained mixture was of a total mass of PS and ZnO 1 g for each sample. To obtain the nanocomposite films, the mixture was dispensed onto a petri dish and kept under vacuum at RT for one week for drying and/or getting rid of excess amounts of the DMF solvents. Homogenous PS/ZnO nanocomposite films of ≈ 0.2 mm thickness were obtained. The ZnO nanocrystal concentrations were varied by 0, 0.5, 0.7, 1.0, and 3.0 wt% to obtain different nanocomposite films. The samples were labeled according to 0, 0.5, 0.7, 1.0, and 3.0 wt% percentages, as PS0, PS0.5, PS0.7, PS1.0 and PS3.0, respectively. ZnO nanocrystals were obtained after calcination that was done at 600 °C for 6 h, The structure of the obtained final product of ZnO nanopowders was examined and confirmed by X-ray diffraction (XRD) using -ray diffractometer (PANalytical X’Pert PRO MRD X with Cu Kα radiation source (PANalytical Inc., MA, USA), field emission scanning electron microscope and Fourier transform infrared (FTIR) according to a previous study [8]. The thermogravimetry (TG) analysis for pure PS and the PS/ZnO nanocomposite films were carried out using a Shimadzu TGA-50H instrument. A mass of about 2 mg of the under-investigation sample was uploaded into the TG platinum pan. A TG heating rate of 10 °C min^−1^ under nitrogen (N_2_) atmosphere and with a flow rate of 30 mL min^−1^ conditions was applied. The TG measurements were done in the temperature range from RT to 600 °C. The differential scanning calorimetric (DSC) measurements were done using DSC instrument (a SETARAM LabsysTM TG-DSC16 system, setaram instrumentation, France). For this cause, DSC alumina crucibles were charged with 20 mg of the sample under examination. The DSC measurements were done under non-isothermal conditions through a constant heating rate of 10 °C /min under an argon (Ar) gas atmosphere. The Ar gas dynamic flow of 25 mL min^−1^ in the temperature range from RT up to 600 °C was used. Scanning electron microscopy (SEM) images were acquired using FE-SEM, (a Quanta FEJ2electron microscope, ThermoFisher Scientific Inc., Waltham, MA, USA). The morphology features and agglomeration rate of the ZnO nanoparticles were characterized by using a high-resolution transmission electron microscope (HR-TEM) (JEM-2100, JEOL Ltd., Tokyo, Japan). The elemental analysis of the prepared nanocomposite samples was examined by an energy dispersive X-ray spectroscopy (EDX) unit that attached to the Quanta FEJ2 electron microscope.

## 3. Results and Discussion

**Figure 1:**
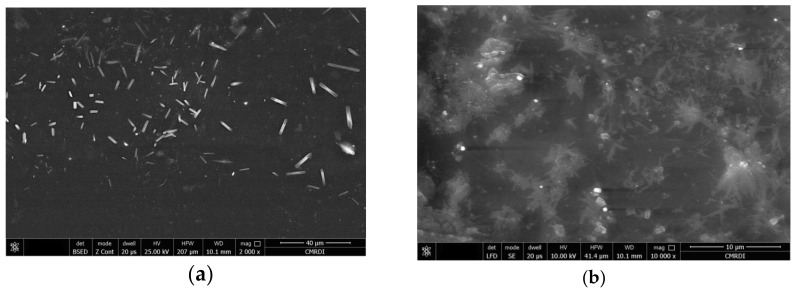
SEM images for PS3.0 sample at (**a**) 2000× (**b**) 100,000× magnifications.

**Figure 2:**
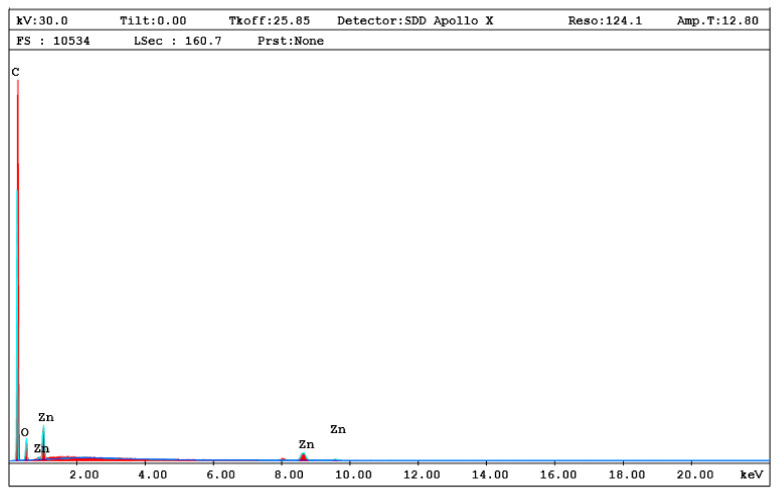
EDX analysis elemental analysis of the rod-like shape features PS3.0 of PS/ZnO nanocomposites.

The morphologies of the PS/ZnO nanocomposite films were examined using the SEM tool. Figure 1 shows SEM images of the PS3.0 sample, as an example for the morphology of SEM images. Profound analysis of the morphology of the SEM image revealed a quite reasonable mixing between PS matrix and ZnO nanorods with some non-completely capped ZnO particles in rod-like shape on the surface of the film as shown in Figure 1a. The existence of the particles on the surface can be attributed to the high concentration of ZnO on this sample. Moreover, Figure 1b reveals the formation of micron-sized agglomerations from ZnO nanorods. SEM confirms the existence of various dispersed ZnO nanorods that enabled a reasonable density of interfacial zones between the nanoparticles and polymer matrix. The elemental analysis in the specific regions of the rod-like shapes was examined using EDX as shown in Figure 2. The existence of Zn, O and C elements in the investigated regions are clearly observed in this figure. This result is considered as evidence for the even dispersion of the ZnO nano-rods in the PS matrix. Such fine dispersion of the nanoparticles could be causing the enhancement of the thermal stability of the investigated PS/ZnO nanocomposite and it will be discussed afterwards.

**Figure 3:**
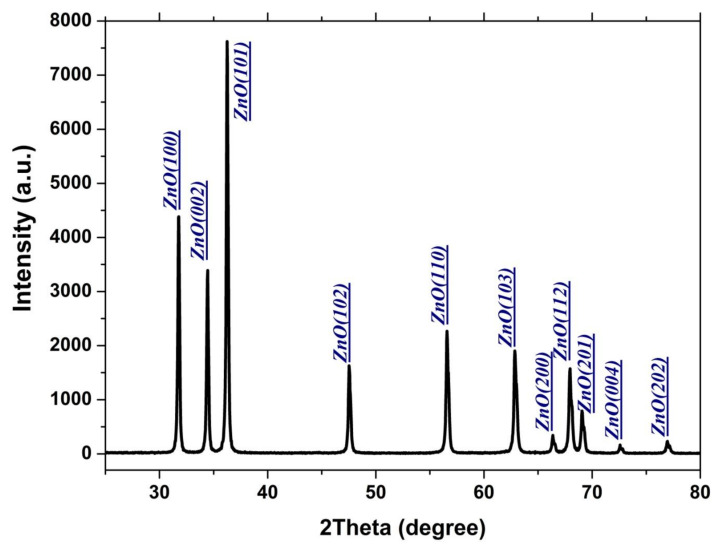
XRD diffrasction pattern of as-synthized ZnO nanoparticles used as PS nanofillers.

Structural characterizations of ZnO nanorods were done using the XRD. Figure 3 shows the XRD pattern of the as-synthesized ZnO nanorods sample that was used as nanofiller in all PS samples with peaks identification on it. ZnO nanorods as seen from XRD patterns are crystallized in a typical wurtzite hexagonal structure for ZnO along (101) direction on the c-axis as a preferential growth direction. Hence all presented XRD peaks in the pattern are identical to that of the ZnO wurtzite structure. No other peaks were identified in the pattern for any other ZnO phase which confirms the single phase of as-synthesized ZnO sample. An average crystallite size (D) of about 39 nm using Scherer formula $D=\frac{0.94\;\lambda }{W_{hkl}\cos\theta }$, where *λ =* 1.5406 Å is the wavelength of used X-ray beam, *W_hkl_* is the full width at half maximum (FWHM) of the XRD peaks, and θ  is the diffraction angle. The lattice constants of the wurtzite unit cell, c =5.2060 Å and a = 3.2462 Å with c/a = 1.603 and unit cell volume of 47.51 Å^3^, were obtained which are matching that of the standard ZnO wurtzite unit cell [26].

**Figure 4:**
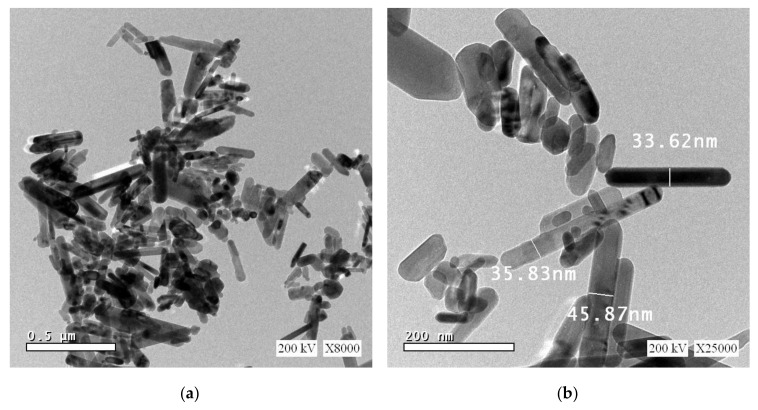
HR-TEM microgrph imges of as-synthized ZnO nanoparticles that used as PS nanofillers at two different mganfications, (**a**) 8000× and (**b**) 25,000×.

Figure 4 shows HR-TEM images for the as-synthesized ZnO nanorods. As can be seen from TEM the micrograph images reveal the formation of ZnO nanorods with some agglomeration as seen in the lower magnification TEM image of Figure 4a. Small nanosized rods are tending to agglomerate as a result of higher surface energy as well as their high surface to volume ratio.

The obtained ZnO nanorods showed good dispersion and distribution to be used as nanofillers in the PS polymer matrix. Figure 4b depicts a higher magnification TEM image for as-synthesized ZnO nanrods. Figure 4b reveals that the sample refers to well-defined rod structures with mixed characteristic sizes. The values that marked the average diameter for the obtained nanorods are also shown in Figure 4b.

**Figure 5:**
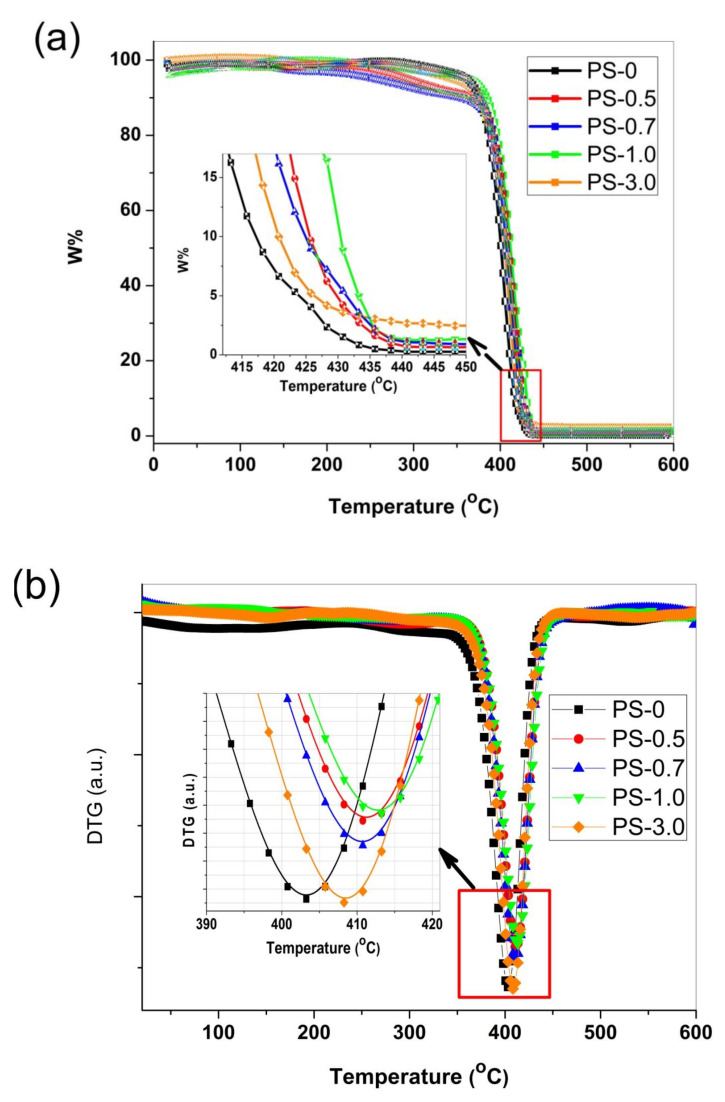
(**a**) TG curves for pure PS and PS/ZnO nanocomposites done at 10 °C min^−1^ heating rate under N_2_ atmosphere and (**b**) DTG curves for pure PS and PS/ZnO nanocomposites.

The thermal stability of the Zn/PS samples was checked using the thermogravimetry (TG) technique and their Derivative thermogravimetric DTG analysis. Figure 5 shows the TG and obtained DTG curves for pure PS and PS/ZnO nanocomposites under investigation. The TG curves of all the investigated samples were showing a single degradation step, as seen in Figure 5a. The remaining weight after the degradation is systematically increased as the ZnO nanoparticles content in PS nanocomposites was increasing, as shown in the enlargement inset of Figure 5a. Such a result refers to the homogeneity of the obtained nanocomposites. The characterization temperatures at different weight loss percentages of ZnO nanoparticles at T_50_ and T_100_ are summarized in Table 1. T_50_ and T_100_ are temperatures at which 50 and 100% of weight losses occurred. The characteristic temperatures show that the thermal stability of all the ZnO nanocomposites is higher compared to that of the pure PS sample. The values of the degradation characterization temperatures that are listed in Table 1 are generally showing a trend of increasing as ZnO content increases in the samples. This is confirming that the ZnO addition is enhancing the thermal stability of PS within the investigated concentration ZnO nanoparticles range. The delay in the polymer degradation as the ZnO nanoparticle content increases in the samples can be explained in view of the decreasing in the chains’ mobility with dispersion of nanoparticles into the polymer matrices [27], since inorganic nanoparticle fillers are non-degradable material with very high surface areas. The high dispersion of the nanoparticles into the polymer matrix is an obstacle for the diffusion of the free radical and as a result a retarding in degradation of the nanocomposites is obtained [10]. Similar results were obtained for PS-based nanocomposites with various nanofillers as nanosilica [28,29,30] and carbon nanoparticles [5] as well as ZnO [10,11]. The delay in the thermal degradation of such investigated nanocomposites was attributed to fine dispersions of used nanofillers. In addition, the high heat capacity and good thermal conductivity of ZnO nanoparticles made them act as good heat sinkers that overcame the amount of heat that the PS polymer backbone could gain and hence enhancement in thermal stability of the composites is attained [25].

Figure 5b shows the obtained DTG curves of all PS/ZnO nanocomposites. For clear observation of DTG peak positions and their shifts, the enlargement of the DTG spectra are depicted for PS and PS/ZnO nanocomposites as an inset of Figure 5b. In addition, it can be seen from Figure 5 that the peak position (T_p_) as well as the characterization temperatures of decomposition (T_50_ and T_100_) for all PS/ZnO nanocomposites showed shifts to higher temperatures as compared to that of pristine PS. Such shifts are clear indications of the enhancement in the thermal stability of the polymer nanocomposites per addition of the ZnO nanoparticles into the polymer matrix. Similar results were reported for PS/ZnO nanocomposites [31]. The thermal stability behavior for these nanocomposites may be due to two overlapped stages of degradation, The T_p_ values of the samples showed an increase from 403.3 °C for PS sample to 413.2 °C for PS1.0 sample. Table 1 illustrates the obtained T_p_ values for PS/ZnO nanocomposites.

For more investigation into the behavior of degradation for Ps/ZnO nanocomposites in comparison with the pristine PS, the Coats-Redfern method was used for activation energy calculations [32,33]. In this method single heating rate data can be used for activation energy. TGA data of the under-investigation PS/ZnO nanocomposites were used for this purpose. In this approach the constant TGA heating rate of weight loss was required [34]. In our calculations we used the Coats-Redfern method [33]:

(1) $$\ln\left(-\frac{\ln{\left(1-\alpha \right)}^{n}}{T^{2}}\right)=\ln\;\left[\frac{\mathrm{AR}}{{\beta E}_{a}}\left(1-\frac{2\mathrm{RT}}{E_{a}}\right)\right]-\frac{E_{a}}{\mathrm{RT}}\;$$

where α is the degradation fraction, n is the reaction order, T is absolute temperature, A is pre-exponential factor, β is constant heating rate, R is gas constant and E_a_ is the activation energy of decomposing. The degradation fraction, α, was obtained from [5,35]:

(2) $$\alpha =\;\frac{W_{\circ }-W_{T}}{W_{\circ }-W_{\infty }}$$

where W_o_, W_T_ and W_∞_ are remaining weights at the initial stage of the degradation process at temperature T and at the reaction end point, respectively.

**Figure 6:**
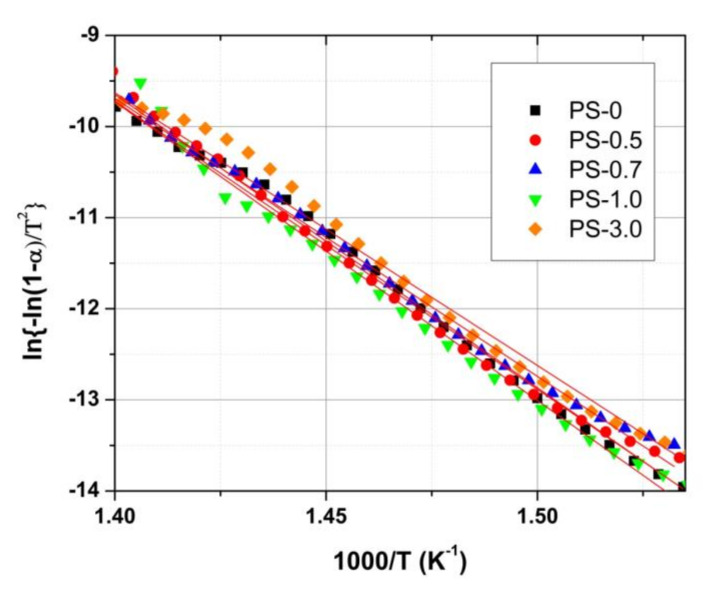
Plots of ln{−ln(1−α)/T^2^} against 1/T of Coats-Redfern equation from TGA data to obtain thermal degradation activation energies of PS/ZnO nanocomposites. Straight lines are fitting lines.

Using the above equations for each TGA curve of PS/ZnO nanocomposites at heating rate, β = 10 °C min^−1^, and reaction order, n is assumed to be a reasonable assumption for many decomposing polymers [36]. The activation energies of decompositions can be obtained from a plot of the left side of equation (1) against 1/T and the slope of obtained straight lines for each sample is used to get activation Energy. Figure 6 shows the plot of plots of ln{−ln(1−α)/T^2^} against 1000/T of the Coats-Redfern equation. The calculated activation energies of PS/ZnO nanocomposites are enlisted in Table 2. The obtained value of the activation energy for pristine PS sample is 263.0 kJ/mole and this is in good agreement with that reported before [35].

The obtained Ea values are 263.0, 267.6, 253.7, 272.6 and 248.8 kJ/mol for PS-0, PS-0.5, PS-0.7, PS-1.0 and PS-3.0 samples respectively. The activation energy of degradation for both PS-0.5 and PS- 1.0 nanocomposites is enhanced by about 3–5 kJ/ mol in comparison with the pristine PS, due to more thermal stability of the nanocomposite samples.

**Figure 7:**
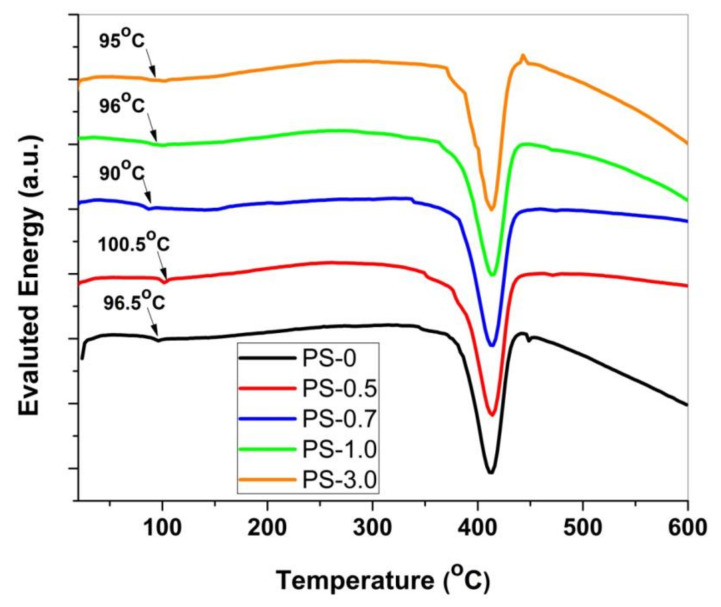
DSC curves were done at non-isothermal conditions of heating rate 10 °C/min under a dynamic flow of Ar gas with a flow rate of 25 mL min^−1^.

The differential scanning calorimetry (DSC) curves for pure PS and PS/ZnO nanocomposites that are done at a heating rate of 10 °C/min are shown in Figure 7. Endothermic peaks which are related to the melting process of the samples are prominent in each curve for all the investigated samples. The obtained values of the melting endothermic temperature peak (T_m_) are also listed in Table 1. The highest value of T_m_ was recorded for PS-1.0 sample. The values of T_p_ are slightly varied with the variation of the ZnO content in the studied PS/ZnO nanocomposites. Such results are in good agreement with that attained for other degradation characterization temperatures (T_50_ and T_100_). Therefore, PS-1.0 is considered the most stable sample of all studied nanocomposites. Here it is worth mentioning that the reduction in the values of all degradation characteristic temperatures for the highest ZnO wt% nanocomposite (PS-1.0) can be attributed to the increase of the probability of the agglomeration of nanoparticles. Such agglomeration has adverse effects on the thermal stability of the investigated nanocomposites due to the expected conversion from nanostructure to microstructure of the ZnO particles. So, the effect of the loaded nanoparticles (1 wt%) on protection of the macromolecular chains from thermal decomposition are reduced in comparison to other studied samples with lower concentrations.

Values of the glass transition temperature, T_g_, of PS/ZnO nanocomposites with change in the wt% of ZnO rod-like nanoparticles, had a non-monotonic trend as shown in Figure 7. In fact, T_g_ values did not show a unique trend with increasing of the ZnO nanofiller in the polymer nanocomposites. This is due to the competition of many factors which affects the degree of freedom of the polymer chains [37]. In most cases of nanocomposites, there are two main competitive factors. One of them is coating the existence organic layer, i.e., polymer, on the surface of nanoparticles which is leading to an increase in the degree of freedom and the entropy of the nanocomposite system and consequently the T_g_ of nanocomposite decreased. The other factor, the presence of inorganic nanoparticles in the polymer matrix, is leading to a reduction in the density of configuration states of the macromolecules that enables the reduction of the disorientation entropy resulting in an increase in T_g_ value. Here it is worth mentioning that the agglomeration of nanoparticles, the shape of the nanoparticles, and the degree of film homogeneity could be varied from one sample to another, especially when using the nanoparticles without any surface treatment or coupling agent. So, such variations in the investigated samples lead to a non-weighting factor which influenced in T^g^ behavior. The increased value of T_g_ in 0.5 wt % sample could be due to good confinement of the PS chains near the ZnO rather than affect by the interaction between PS and ZnO nanorods [10].

DSC results in Figure 7 also showed the presence of endothermic peaks in all samples around 440–450 °C which are obvious indications of the competence of the decomposition of PS/Zno nanocomposites. Also, very small endothermic peaks were obtained at a low temperature range close to 100 °C. The endothermic peak at this temperature value is for the water vapor. No other pronounced thermal peaks were observed besides the fundamental endothermic peaks. This result confirmed the formation of nanocomposites with high thermal stability as well as being in agreement with TG measurements that the degradation occurs via a single-step process.

## 4. Conclusions

PS/ZnO nanocomposites loaded with ZnO nanorods in the range 0–3 wt% were obtained. XRD and TEM analyses affirmed the formation of ZnO nanorods with a good dispersion and matching the standard ZnO wurtzite structure. The thermal stability of the resulted PS/ZnO nanocomposites was very good because of the addition of ZnO nanorods in the investigation range. Tg increases on addition of ZnO by 0.5 wt% (PS-0.5) by 4 °C nanocomposites compared to pristine PS, then it remains nearly unchanged in the vicinity of 96 °C. The TG, DTG and DSC resulting data are referring to the most thermally stable nanocomposite obtained at 1 wt% of ZnO nanorods (PS-1). Moreover, all used concentration of ZnO nanorods have a positive effect on delaying the degradation process of the PS polymer chains as confirmed from the increasing in the T_50_ and T_100_ from TGA measurements for pristine PS and PS-1 from 401 and 440 °C to 412 and 451 °C, respectively. This is confirmed in the high dispersion of the ZnO nanorods in PS matrix. The degradation effect of ZnO nanorods was explained in view of the increase in the surface area of non-degradable material throughout the polymer matrix as ZnO content increases. In summary, the improvement of the thermal stability of PS/ZnO nanocomposites is confirmed by activation energy calculations. Activation energies that were obtained from TGA data at constant heating rate are showing small enhancement by 3–9 kJ/mole after the addition of ZnO nanorods. The role of used ZnO nanofillers is as an efficient heat sinker as well as a protective barrier for degradation process of polymer chains.

## Figures and Tables

**Table 1:** Characteristic temperatures, T_50%_ and T_100%_ T_m_ and T_p_ are temperatures at which 50% and 100% weight loss of ZnO nanoparticles occurs, endothermic temperature peak and peak position, respectively.

Sample	T_50_ °C	T_100_ °C	T_P_ °C	T_m_ °C
PS-0	401	440	403.3	412.3
PS-0.5	409.3	441	410.8	413.5
PS-0.7	409	445	410.7	413.5
PS-1.0	412	451	413.2	414.4
PS-3.0	405.5	445	408.3	412.8

**Table 2:** Thermal degradation activation energies of pristine PS and PS ZnO nanocomposites obtained from Coats-Redfern method.

Sample	Ea (kJ/mol)	R^2^
PS-0	263.0	0.997
PS-0.5	267.6	0.996
PS-0.7	253.7	0.998
PS-1.0	272.6	0.995
PS-3.0	248.8	0.992
